# Prognostic value of EBV DNA and platelet-to-lymphocyte ratio in patients with non-metastatic nasopharyngeal carcinoma: a retrospective study

**DOI:** 10.1186/s12885-023-11117-5

**Published:** 2023-07-18

**Authors:** Huan Dong, Zichong Huang, Dong Yang, Zhiru Li, Heqing Huang, Zhen Meng, Yutao Qin, Min Kang

**Affiliations:** 1 Department of Radiation Oncology, The First Affiliated Hospital of Guangxi Medical University, No. 6, Shuangyong Road, Nanning, Guangxi, 530021 People’s Republic of China; 2 Department of Radiotherapy and Chemotherapy, The Second People’s Hospital of Yichang, No. 21, Xiling 1st Road, Yichang, Hubei, 443000 People’s Republic of China; 3 Department of Oncology, Langdong Hospital of Guangxi Medical University, No. 60, Jinhu North Road, Nanning, Guangxi, 530028 People’s Republic of China

**Keywords:** Nasopharyngeal carcinoma, Prognosis, Platelet-to-lymphocyte ratio, Epstein-Barr virus DNA

## Abstract

**Purpose:**

Analyzing the prognostic value of Epstein-Barr virus (EBV) DNA load and platelet-to-lymphocyte ratio (PLR) in non-metastatic nasopharyngeal carcinoma (NPC) patients, thereby developing a reliable and effective marker.

**Methods:**

We compared survival rates among different groups using the Kaplan-Meier method and the Log-rank test. The factors affecting the prognosis of NPC patients were determined using univariate and multivariate cox regression analysis. Receiver operating characteristic (ROC) curves were used to identify the cutoff-value and discriminant performance of the model.

**Results:**

The ROC curve indicated a cut-off value of 775 copies/ml for EBV DNA and 203.3 for PLR. Kaplan-Meier and Log-rank tests showed that 3-year overall survival (OS), local recurrence-free survival (LRFS) and distant metastasis-free survival (DMFS) of NPC patients in high risk group (HRG) were significantly poorer than those in medium risk group (MRG) and low risk group (LRG). The 3-year OS of NPC patients was significantly correlated with age, N stage and EBV DNA-PLR. The 3-year LRFS were significantly correlated with sex, N stage, histology type, and EBV DNA-PLR. The 3-year DMFS were correlated with histology type. The ROC curve showed that area under the curve (AUC) values of EBV DNA-PLR of 3-year OS, LRFS and DMFS in NPC were higher than those of PLR and EBV DNA.

**Conclusion:**

EBV DNA-PLR is an independent risk factor for the prognosis of NPC. Compared with PLR or EBV DNA alone, the combination of EBV DNA and PLR may be more accurate in predicting the prognosis of NPC patients.

## Introduction

In southern China and Southeast Asia, nasopharyngeal carcinoma (NPC) is a common malignant tumor of the head and neck. It has obvious geographical distribution characteristics. Most of its histology types are non-keratinizing carcinoma. NPC lacks specific symptoms in the early stage, and most of them are diagnosed in the late stage. While the pathogenesis of NPC remains unclear, most studies have demonstrated that genetic susceptibility, eating habits, and Epstein-Barr virus (EBV) infection are risk factors [1]. Because the location of NPC is deep and special, cervical lymph node metastasis usually occurs, the effect of operation is poor and difficult, and NPC usually has high radiosensitivity, so radiotherapy is the main treatment of it. In addition, the combination of radiotherapy and chemotherapy is more effective in the treatment of advanced NPC [2]. In the present, the TNM staging system proposed by the International Union Against Cancer/American Joint Committee on Cancer is mainly used to guide clinical management and prognosis of NPC [3, 4]. However, it has been found that this staging system could not always accurately predict the prognosis of patients with NPC. Although patients at the same stage and receiving the same treatment, more than 20% of the patients have poor efficacy, which is due to the defect of the prognosis evaluation of the TNM staging system, which is difficult to reflect the biological behavior and immune heterogeneity of tumors [5–8]. Therefore, in addition to improving the treatment of NPC, looking for reliable and economical prognostic indicators to evaluate the prognosis more accurately is also a necessary condition for us to determine the malignant degree of NPC and optimize the treatment.

Many blood markers have been considered prognostic markers for NPC patients in recent years, including Epstein-Barr virus DNA (EBV DNA) [9], hemoglobin [10], albumin [11], C-reactive protein (CRP) [12] and lactate dehydrogenase (LDH) [13]. There is evidence that EBV DNA can be measured before treatment in most NPC patients, so plasma free EBV DNA load is the most important biomarker to reflect the tumor load of NPC [1, 14]. Due to the different sensitivity of each research center to EBV DNA, there is no unified conclusion on the cut-off value of high or low expression of EBV DNA, and it seems difficult to evaluate the biological characteristics and heterogeneity of NPC patients only on the basis of TNM stage or EBV DNA. Complete blood count is one of the most common laboratory testing methods. Many observational and experimental studies have confirmed that abnormal complete blood count is related to the occurrence and development of tumors. Anemia is closely linked to the prognosis of colorectal cancer and endometrial cancer [15, 16]. Tumor development is influenced by the immune status of the body as well [17]. Lymphocytes are the most important effector cells in tumor immune response. Different types of tumors are significantly associated with inflammatory markers such as neutrophil-to-lymphocyte ratios (NLR) and platelet-to-lymphocyte ratios (PLR) [18, 19]. Numerous studies have shown that PLR plays a key role in NPC prognosis in recent years. Therefore, our study combined pre-treatment EBV DNA load with pre-treatment PLR to explore its correlation with general clinical features, survival and prognosis of NPC patients, and to analyze its discriminant performance for prognosis of NPC patients.

## Methods

### Clinical subjects

One hundred and ninety-eight NPC patients treated in the Radiotherapy Department of the First affiliated Hospital of Guangxi Medical University from November 2017 to August 2019 were retrospectively analyzed for this study. Inclusion criteria for our study were as follows: (1) NPC patients was diagnosed by histology; (2) there was no distant metastasis before or during treatment; (3) had not received any antineoplastic therapy in the past; (4) denied the history of other malignant tumors; (5) physical status score 0–1 for Eastern Cooperative Oncology Group; (6) received radiotherapy or concurrent chemoradiothrapy with / without induction or adjuvant chemotherapy, and completed the entire treatment. (7) clinical data, examination data and follow-up data were available. The exclusion criteria were: (1) distant metastasis was found before or during treatment; (2) serious complications; (3) pregnant or lactating women; (4) previous or simultaneous occurrence of other malignant tumors; (5) unable to complete the treatment. This study was conducted in accordance with the Helsinki Declaration and approved by the Ethics Committee of the First Affiliated Hospital of Guangxi Medical University. And this was a retrospective study, so the informed consent was waived by the Ethics Committee of the First Affiliated Hospital of Guangxi Medical University. Participant information is confidential.

### Clinical data collection

The data of the following variables were recorded by consulting medical records: sex, age, histology, TNM stage, smoking history, family history, treatment, complete blood count results and EBV DNA copy number. In the study, PLR was used, and the PLR is calculated as the platelet count per 10^9^/L divided by the lymphocyte count per 10^9^/L.

### Therapeutic schedule

In accordance with National Comprehensive Cancer Network guidelines, a standardized treatment plan was established according to the TNM stage of the patients. Stage I patients received radical radiotherapy. Stage II patients received radiotherapy or concurrent chemoradiothrapy combined with platinum drugs. Stage III-IVa patients received concurrent with induction chemotherapy or adjuvant chemotherapy. The patients were all treated with intensity modulated radiotherapy (IMRT). The radiotherapy target areas of NPC include gross tumor volume of nasopharynx (GTVnx), metastatic cervical lymph node volume (GTVnd), surrounding subclinical area (clinical target volume 1, CTV1) and cervical lymphatic drainage area (CTV2) need prophylactic irradiation. Regarding the total prescribed dose, GTVnx was 68 ~ 76 Gy / 30 ~ 33 f, GTVnd was 66 ~ 70 Gy / 30 ~ 33 f, CTV1 was 60 ~ 64 Gy / 30 ~ 33 f;CTV2 was 50 ~ 54 Gy / 30 ~ 33 f. The fractional dose was 2.00 ~ 2.33 Gy/f. Induction or adjuvant chemotherapy included GP regimen, TPF regimen, PF regimen and TP regimen. The GP regimen included gemcitabine doses of 1000 mg/m^2^ on days 1 and 8 and cisplatin doses of 80 mg/m^2^ on day 1. The TPF regimen consists of docetaxel 60 mg/m^2^ on day 1, cisplatin 60 mg/m^2^ on day 1, and continuous intravenous drips of 5-fluorouracil 600 mg/m^2^ from day 1 to day 5. The PF regimen consisted of 80 mg/m^2^ of cisplatin on day 1, 800 ~ 1000 mg/m^2^ of 5-fluorouracil, continuous intravenous drip from day 1 to day 5. In the TP regimen, docetaxel was administered at 75 mg/m^2^ on day 1 and cisplatin was administered at 75 mg/m^2^ on day 1. A total of 2–3 cycles of each regimen were performed every 21 days. Concurrent chemotherapy regimen was mainly cisplatin (80 ~ 100 mg/m^2^, every 21 days for a total of 2–3 cycles; 30 ~ 40 mg/m^2^, every 7 days for a total of 5–6 cycles). In the case of patients who were not suitable for cisplatin, other platinum drugs were used instead.

### Endpoint and follow-up

Overall survival (OS) was our primary endpoint, followed by distant metastasis-free survival (DMFS) and local recurrence-free survival (LRFS). Generally, the OS is the duration of treatment between the onset of treatment and the death due to any cause. In this study, LRFS was calculated from the beginning of treatment to the first occurrence of local recurrence. The DMFS is calculated from the start of treatment until distant metastases are detected. Patients were followed up every 3 months for the first 2 years after treatment, every 6 months for 3-5-year after treatment, or until death. In follow-up, a magnetic resonance imaging scan of the nasopharynx and neck should be performed, as well as a computed tomography scan of the chest and abdomen. To confirm local recurrence or distant metastasis, fine needle puncture or pathological tissue biopsy should be performed if necessary. Patients who were lost to follow-up or were still alive without distant metastasis or locoregional recurrence at the end of the trial had their data censored at the date of last follow-up.

### Statistical analysis

SPSS version 26.0 (IBM Corporation, Armonk, NY, USA) and MedCalc 20.1 (MedCalc Software Ltd, Ostend, Belgium) were used for all statistical analyses. The general characteristics of patients were compared with frequency and descriptive statistics. A chi-square test or Fisher’s exact test was used to compare the characteristics of patients in different groups. To determine the cut-off value of the research indicators based on 3-year OS, Youden index of receiver operating characteristic (ROC) curves were used. Then, the research indicators were divided into high or low according to the cut-off value. To plot survival curves and compare survival among groups, Kaplan-Meier and Log rank tests were used. The factors with P < 0.2 were selected for multivariate analysis based on univariate cox regression analysis. The multivariate cox regression analysis showed independent risk factors for NPC with P < 0.05. A ROC curve was performed to assess whether NPC prognosis could be accurately predicted by EBV DNA combined with PLR. The area under the curve (AUC) > 0.6 is considered to be a predictive value. Delong test was used to compare the classification efficiency of these ROC curves. P < 0.05 indicated a important statistical significance.

## Results

### EBV DNA-PLR combined score

According to the ROC curve, the cut-off values of EBV DNA and PLR were 775 (copies/ml) and 203.3, respectively. EBV DNA < 775 was considered as low, EBV DNA > 775 was considered as high, and PLR < 203.3 was regarded as low, PLR > 203.3 was regarded as high. To explore the prognostic value of EBV DNA and PLR, we established the EBV DNA-PLR combined score, which is a new prognostic factor based on EBV DNA. Based on the score, patients are divided into three groups: low risk group (LRG), medium risk group (MRG), and high risk group (HRG). The EBV DNA-PLR scoring criteria and grouping were as follows: high EBV DNA and high PLR as high risk group with 2 score, high EBV DNA and low PLR or low EBV DNA and high PLR as medium risk group with 1 score, and low EBV DNA and low PLR as low risk group with 0 score.

### Patient characteristics

The median age of 198 NPC patients in our study was 46 years (range 16–73 years), including 140 (70.7%) males and 58 (29.3%) females. In terms of TNM staging, there were 55(27.8%) in stage T1 ~ T2, 74 (37.4%) in stage T3, 69 (34.8%) in stage T4, 87 (43.9%) in stage N0 ~ N1, 76 (38.4%) in stage N2, 35 (17.7%) in stage N3, 111 (56.1%) in stage I ~ III, 87 (43.9%) in stage IVa. In the light of histology, a total of 187 (94.4%) were undifferentiated non-keratinizing carcinomas (WHO histology type III) and 11 (5.6%) were differentiated non-keratinizing carcinomas (WHO histology type II). Among these NPC patients, 17 (8.6%) patients received radiotherapy alone, 93 (47.0%) patients received concurrent chemoradiothrapy, and 88 (44.4%) patients received concurrent chemoradiothrapy plus induction chemotherapy or adjuvant chemotherapy (Table 1).

**Table 1:** NPC patients’ characteristics divided by EBV DNA-PLR combined score

Characteristics	Total(%)	LRG(%)	MRG(%)	HRG(%)	*p*-Value
Sex					0.483^a^
Male	140(70.7)	66(75.0)	57(67.9)	17(65.4)	
Female	58(29.3)	22(25.0)	27(32.1)	9(34.6)	
Age(years)					
< 46	89(44.9)	49(55.7)	35(41.7)	5(19.2)	**0.003** ^a^
≥ 46	109(55.1)	39(44.3)	49(58.3)	21(80.8)	
T stage					**0.006** ^a^
T1-T2	55(27.8)	34(38.6)	18(21.4)	3(11.5)	
T3	74(37.4)	31(35.2)	35(41.7)	8(30.8)	
T4	69(34.8)	23(26.1)	31(36.9)	15(57.7)	
N stage					**0.001** ^a^
N0-N1	87(43.9)	50(56.8)	30(35.7)	7(26.9)	
N2	76(38.4)	31(35.2)	31(36.9)	14(53.8)	
N3	35(17.7)	7(8.0)	23(27.4)	5(19.2)	
Overall stage					**0.003** ^a^
I-III	111(56.1)	60(68.2)	42(50.0)	9(34.6)	
IVa	87(43.9)	28(31.8)	42(50.0)	17(65.4)	
Histology type					0.909^b^
II	11(5.6)	6(6.8)	4(4.8)	1(3.8)	
III	187(94.4)	82(93.2)	80(95.2)	25(96.2)	
Smoking					0.343^a^
No	122(61.6)	57(64.8)	47(56.0)	18(69.2)	
Yes	76(38.4)	31(35.2)	37(44.0)	8(30.8)	
Family history					0.246^a^
No	182(91.9)	79(89.8)	77(91.7)	26(100.0)	
Yes	16(8.1)	9(10.2)	7(8.3)	0(0.0)	
pre-EBV DNA					**0.001** ^a^
Negative (< 400)	96(48.5)	70(79.5)	26(31.0)	0(0.0)	
Positive (> 400)	102(51.5)	18(20.5)	58(69.0)	26(100.0)	
Treatment					0.597^a^
IMRT	17(8.6)	10(11.4)	4(4.8)	3(11.5)	
CCRT	93(47.0)	39(44.3)	42(50.0)	12(46.2)	
CCRT + IC/AC	88(44.4)	39(44.3)	38(45.2)	11(42.3)	

Notes: Bold indicates a significant difference among groups with *p* < 0.05

^a^Chi-square test

^b^Fisher’s exact test

Abbreviations: IMRT, intensity-modulated radiotherapy; CCRT, concurrent chemoradiotherapy; IC, induction chemotherapy; AC, adjuvant chemotherapy. LRG, low risk group; MRG, medium risk group; HRG, high risk group

### Survival of different groups in EBV DNA-PLR

**Fig. 1:**
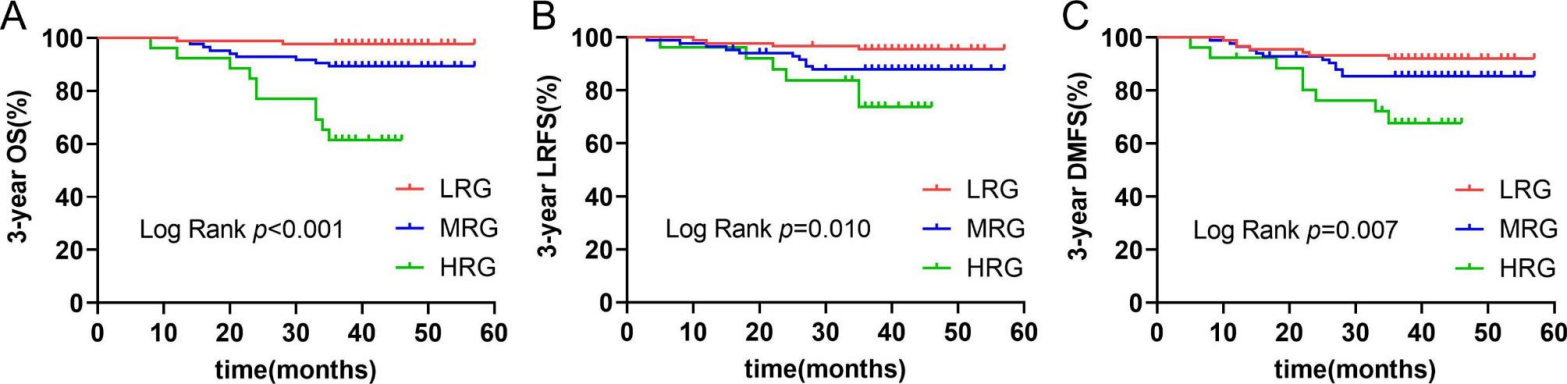
Kaplan-Meier survival curves of 3-year OS (**A**), LRFS (**B**) and DMFS (**C**) in LRG, MRG and HRG. Abbreviations: OS, overall survival; LRFS, local recurrence-free survival; DMFS, distant metastasis-free survival; LRG, low risk group; MRG, medium risk group; HRG, high risk group

**Fig. 2:**
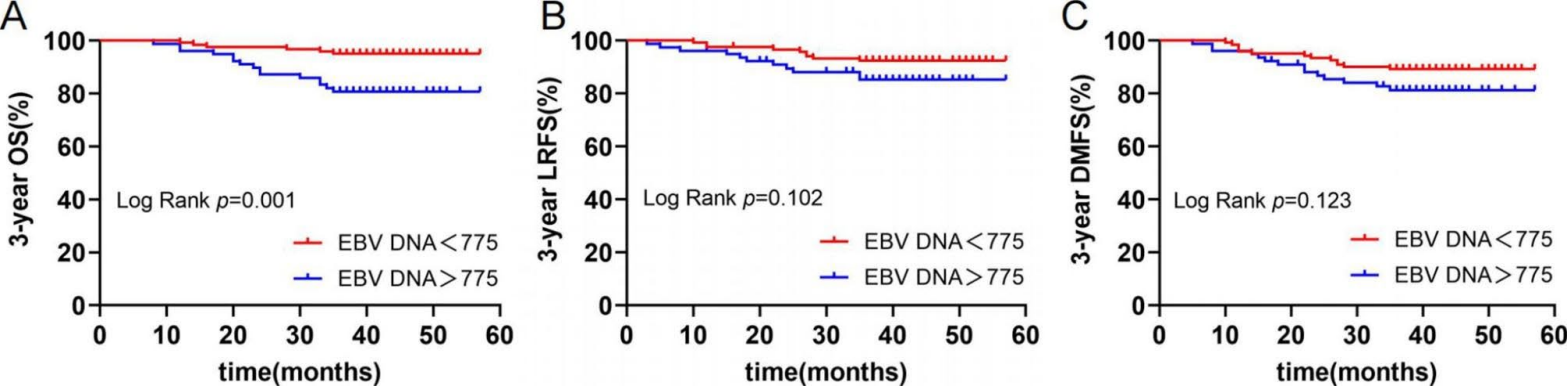
Kaplan-Meier survival curves of 3-year OS (**A**), LRFS (**B**) and DMFS (**C**) in different EBV DNA levels(< 775 vs.> 775) Abbreviations: OS, overall survival; LRFS, local recurrence-free survival; DMFS, distant metastasis-free survival

**Fig. 3:**
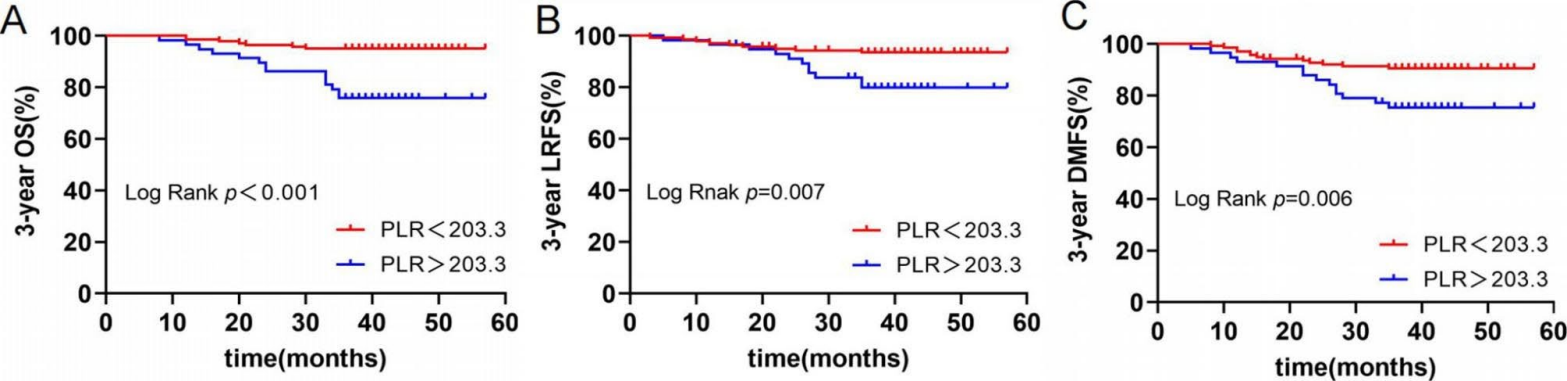
Kaplan-Meier survival curves of 3-year OS (**A**), LRFS (**B**) and DMFS (**C**) in different PLR levels(< 203.3 vs.> 203.3) Abbreviations: OS, overall survival; LRFS, local recurrence-free survival; DMFS, distant metastasis-free survival

The final follow-up was on August 30, 2022, the median follow-up time was 41 months (range 8–57 months). Nineteen of the 198 NPC patients died, 20 had local recurrences, and 27 had distant metastases. The 3-year OS, LRFS and DMFS were 90.4%, 89.9% and 86.4%, respectively. Two patients died, four had local recurrences, and seven had distant metastases in LRG (0 score). Three-year OS, LRFS, and DMFS were respectively 97.7%, 95.5%, and 92.0%. Nine patients died, 10 had local recurrences, and 12 had distant metastases in MRG (1 score). Three-year OS, LRFS and DMFS were 89.3%, 88.1% and 85.7%, respectively. Ten patients died, 6 had local recurrences, and 8 had distant metastases in HRG (2 score). Three-year OS, LRFS and DMFS were 61.5%, 76.9% and 69.2%, respectively. Kaplan-Meier and log-rank tests showed that the 3-year OS, LRFS, and DMFS of NPC patients in HRG were significantly worse than those in MRG and LRG (Fig. 1). The survival curves of patients in three groups are presented in Fig. 1. The survival curves of patients in different EBV DNA (< 775 vs.> 775) and PLR levels (< 203.3 vs.> 203.3) are presented in Figs. 2 and 3, respectively.

### Univariate and multivariate analysis of prognostic factors for NPC

As shown in Table 2, according to univariate cox regression analysis, a number of variables contributed to 3-year OS, including age, EBV DNA-PLR and clinical stage. Variables associated with 3-year LRFS, including sex, age, N stage, histological type, and EBV DNA-PLR. Age, N stage, histological type, and EBV DNA-PLR are variables associated with 3-year DMFS.

**Table 2:** Univariate analysis of prognostic factors in NPC patients

Characteristics	3-year OS		3-year LRFS			3-year DMFS		
	HR(95%CI)	*p*-Value	HR(95%CI)	*p*-Value		HR(95%CI)		*p*-Value
Sex								
Male	Reference		Reference			Reference		
Female	0.944(0.366–2.434)	0.906	2.502(1.041–6.011)		**0.040**	0.998(0.437–2.280)	0.997	
Age(years)								
< 46	Reference		Reference			Reference		
≥ 46	5.221(1.538–17.726)	**0.008**	2.583(0.938–7.108)		**0.066**	2.023(0.777–5.266)	**0.149**	
T stage								
T1-2	Reference		Reference			Reference		
T3	1.145(0.323–4.058)	0.834	1.398(0.464–4.171)		0.548	1.406(0.502–3.802)	0.502	
T4	2.354(0.750–7.394)	**0.143**	1.009(0.308–3.307)		0.988	1.447(0.526–3.983)	0.472	
N stage								
N0-N1	Reference		Reference			Reference		
N2	2.298(0.698–7.632)	**0.174**	1.890(0.618–5.777)		0.264	1.284(0.522–3.160)	0.586	
N3	6.365(1.959–20.678)	**0.002**	4.048(1.284–12.765)		**0.017**	2.548(0.982–6.610)	**0.054**	
Histology type								
II	Reference		Reference			Reference		
III	1.183(0.159–8.813)	0.870	0.319(0.094–1.091)		**0.069**	0.306(0.106–0.885)	**0.029**	
Smoking								
No	Reference		Reference			Reference		
Yes	1.528(0.649–3.597)	0.332	0.875(0.349–2.194)		0.776	1.356(0.635–2.897)	0.432	
Family history								
No	Reference		Reference			Reference		
Yes	0.043(0.000-37.654)	0.363	0.043(0.000-41.142)		0.370	0.043(0.000-15.312)	0.294	
Treatment								
IMRT	Reference		Reference			Reference		
CCRT	1.823(0.233–14.242)	0.567	2.418(0.316–18.486)		0.395	2.826(0.373–21.394)	0.314	
CCRT + IC/AC	1.943(0.249–15.177)	0.527	1.152(0.139–9.570)		0.896	2.152(0.278–16.670)	0.463	
EBV DNA-PLR								
LRG (0 point)	Reference		Reference			Reference		
MRG (1 point)	4.924(1.064–22.792)	**0.041**	2.792(0.876–8.904)		**0.083**	1.885(0.742–4.789)	**0.183**	
HRG (2 point)	19.867(4.349–90.752)	**< 0.001**	5.985(1.687–21.230)		**0.006**	4.503(1.632–12.429)	**0.004**	

Notes: Bold indicates statistically significant with *p* < 0.2

Abbreviations: IMRT, intensity-modulated radiotherapy; CCRT, concurrent chemoradiotherapy; IC, induction chemotherapy; AC, adjuvant chemotherapy; LRG, low risk group; MRG, medium risk group; HRG, high risk group. EBV, Epstein-Barr virus; PLR, platelet-to-lymphocyte ratio; OS, overall survival; LRFS, local recurrence-free survival; DMFS, distant metastasis-free survival; HR, hazard ratio; CI, confidence interval

Then, the above variables were introduced in the multivariate cox regression analysis. As shown in Table 3, significant associations were found between 3-year OS and age (P = 0.048), N stage (P = 0.011) and EBV DNA-PLR (P = 0.002). There were also significant correlations between 3-year LRFS and sex (P = 0.048), N stage (P = 0.043), histological (P = 0.009), and EBV DNA-PLR (P = 0.045). Three-year DMFS was significantly correlated with histological (P = 0.010) and EBV DNA-PLR (P = 0.010).

**Table 3:** Multivariable analysis of prognostic factors in NPC patients

Characteristics	3-year OS		3-year LRFS			3-year DMFS		
	HR(95%CI)	*p*-Value	HR(95%CI)	*p*-Value		HR(95%CI)		*p*-Value
Sex								
Male			Reference					
Female			2.449(1.007–5.955)		**0.048**			
Age(years)								
< 46	Reference		Reference			Reference		
≥ 46	3.681(1.030-13.158)	**0.045**	2.442(0.833–7.161)		0.104	1.222(0.534–2.796)	0.634	
T stage								
T1-2	Reference							
T3	0.497(0.134–1.841)	0.295						
T4	0.741(0.205–2.488)	0.597						
N stage								
N0-N1	Reference		Reference			Reference		
N2	1.544(0.457–5.209)	0.484	1.331(0.425–4.164)		0.624	1.059(0.423–2.648)	0.903	
N3	4.900(1.431–16.786)	**0.011**	3.431(1.038–11.336)		**0.043**	2.259(0.833–6.125)	0.109	
Histology type								
II			Reference			Reference		
III			0.177(0.048–0.652)		**0.009**	1.222(0.534–2.796)	**0.010**	
EBV DNA-PLR								
LRG (0 point)	Reference		Reference			Reference		
MRG (1 point)	3.317(0.694–15.868)	0.133	1.831(0.547–6.128)		0.327	1.579(0.594–4.198)	0.360	
HRG (2 point)	11.897(2.441–57.977)	**0.002**	3.856(1.033–14.395)		**0.045**	4.135(1.401–12.201)	**0.010**	

Notes: Bold indicates statistically significant with *p* < 0.05

Abbreviations: LRG, low risk group; MRG, medium risk group; HRG, high risk group. EBV, Epstein-Barr virus; PLR, platelet-to-lymphocyte ratio; OS, overall survival; LRFS, local recurrence-free survival; DMFS, distant metastasis-free survival; HR, hazard ratio; CI, confidence interval

### Discriminant performance of EBV DNA-PLR to the prognosis

**Fig. 4:**
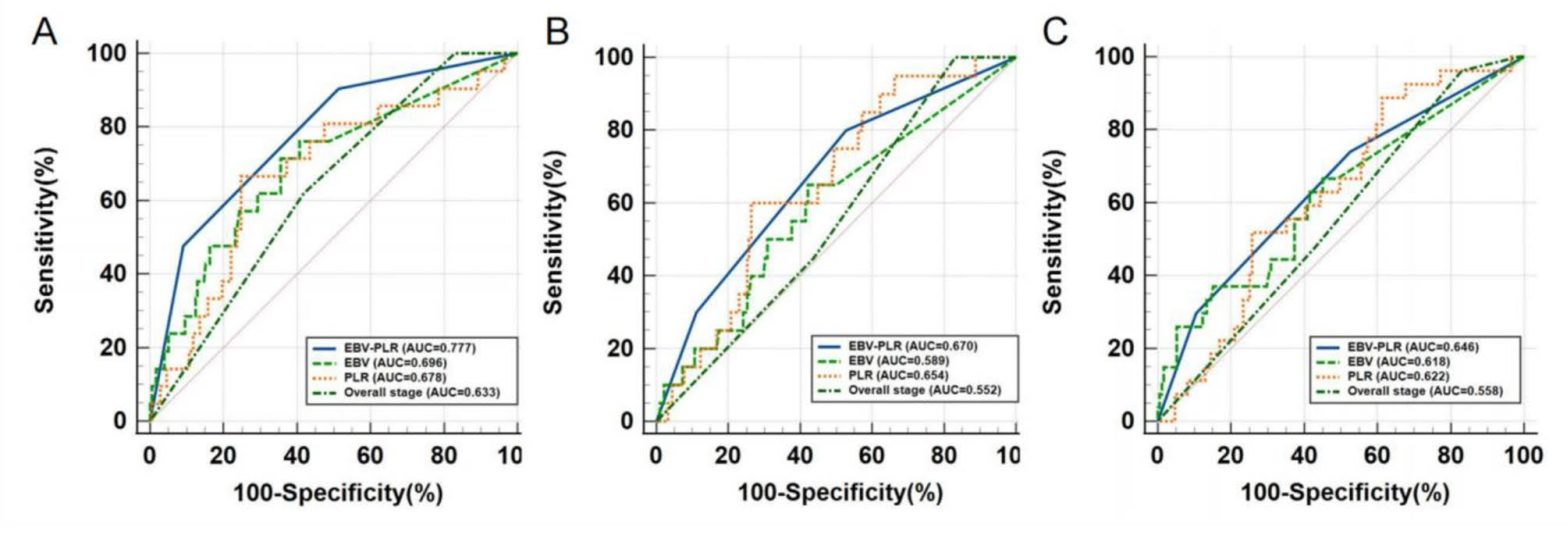
ROC curves for EBV DNA-PLR, PLR, EBV DNA and Overall stage of 3-year OS (**A**), LRFS (**B**), DMFS (**C**) Abbreviations: EBV, Epstein-Barr virus; PLR, platelet-to-lymphocyte ratio; OS, overall survival; LRFS, local recurrence-free survival; DMFS, distant metastasis-free survival; ROC, receiver operating characteristic

Through the analysis of ROC curve, it was concluded that the AUC values of EBV DNA-PLR of 3-year OS, LRFS and DMFS in NPC were higher than those of PLR, EBV DNA and overall stage. There was significant difference between EBV DNA-PLR and overall stage in AUC values of 3-year OS (P = 0.035) (Table 4; Fig. 4).

**Table 4:** Comparison of ROC curves of EBV DNA-PLR, PLR, EBV DNA and Overall stage

Prognostic factors	3-year OS		3-year LRFS		3-year DMFS	
	AUC	*p*-Value	AUC	*p*-Value	AUC	*p*-Value
EBV DNA-PLR	0.777	N/A	0.670	N/A	0.646	N/A
PLR	0.678	0.051^c^	0.654	0.750^c^	0.622	0.608^c^
EBV DNA	0.696	0.112^d^	0.589	0.082^d^	0.618	0.573^d^
Overall stage	0.633	**0.035** ^**e**^	0.552	0.067^**e**^	0.558	0.156^**e**^

Notes: Bold indicates statistically significant with p < 0.05

^c^Comparison between EBV DNA-PLR and PLR.

^d^Comparison between EBV DNA-PLR and EBV DNA.

^e^Comparison between EBV DNA-PLR and Overall stage

Abbreviations: EBV, Epstein-Barr virus; PLR, Platelet lymphocyte ratio; OS, overall survival; LRFS, local recurrence-free survival; DMFS, distant metastasis-free survival; ROC, receiver operating characteristic; AUC, area under the curve

## Discussion

Since the introduction of intensity modulated radiation therapy (IMRT) and immunotherapy, the local control rate of NPC patients has increased considerably. Five-year overall survival rate of NPC patients has reached over 80% [1, 20]. In spite of this, local recurrence and distant metastasis still remain the main causes of failure of treatment [21, 22]. At present, the prognosis of NPC patients still depends on TNM staging system [3]. Nevertheless, although patients have the same clinical stage and receive the same treatment, there are still different treatment effects, which may be due to tumor heterogeneity, immune and inflammatory responses. In fact, tumor immune response plays a major role in the occurrence and progress of various solid malignant tumors [23]. There is also increasing evidence that the inflammatory response plays a key role in tumor development and shows independent prognostic value, such as NLR [18], PLR [19], CRP [12], LDH [13], etc. Inflammatory and immune responses are associated with all stages of tumorigenesis and progression, including initiation, promotion, and metastasis [24].

Tumor growth requires a rich blood supply, and platelets can promote angiogenesis and release growth factors [25]. Tumor cells mediate platelet aggregation [26]. Platelet aggregation around tumor cells protects it from natural killer cell killing [27]. It could be activated by transforming growth factor-β signal transduction pathway regulates the process of tumor micrometastasis and promotes tumor cell exosmosis [28]. Lymphocytes are the most important effector cells in tumor immunity. PLR, as an indicator of the combination of platelet and lymphocyte counts, can reflect the pro-tumor state, inflammatory response and anti-tumor immune state in the body. A meta-analysis has shown that PLR is a risk factor for poor prognosis of various malignant tumors [19]. It has been demonstrated that PLR predicts OS, PFS, LRFS, and DMFS among patients with NPC by Chen et al. and Peng et al [29, 30].

Now many studies have confirmed that plasma EBV DNA load is the most important biomarker reflecting the tumor burden of NPC [14, 31]. However, the sensitivity of detecting EBV DNA load varies among research centers, which may be attributed to different detection reagents or methods. Therefore, there is no generally accepted and uniform EBV DNA cut-off value, and accurate risk stratification cannot be performed based on this indicator. In this research center, when the EBV DNA load is less than 400 copies/ml, it cannot be detected, and we will record the undetectable EBV DNA as 0 copies/ml.

Relying solely on TNM stage and EBV DNA or PLR to evaluate the prognosis is one-sided, which may ignore the effects of tumor load, heterogeneity, immune and inflammatory response on the development of NPC. Therefore, based on the current research results that both EBV DNA and PLR have an impact on the prognosis of NPC, our study combined these two hematological parameters and established EBV DNA-PLR combined score, and divided the NPC patients into groups according to different scores, and to investigate the correlation between hematological parameters and clinical characteristics, survival and prognosis of patients with NPC. In the comparison of patients’ baseline characteristics, it was found that there were significant differences in age, T stage, N stage and overall stage among different groups of NPC patients. In survival analysis, patients in HRG showed significant differences compared with MRG and LRG, NPC patients with HRG had poorer 3-year OS, LRFS and DMFS. Sex, age, EBV DNA-PLR, T stage, N stage, and histology type were included in multivariate analysis based on univariate analysis. Since Overall stage was determined by T stage and N stage, we did not include it in univariate and multivariate analysis. The results of multivariate analysis showed that the EBV DNA-PLR was an independent prognostic factor for NPC patients. There were significant differences in 3-year OS (HR: 11.897, 95% CI: 2.441–57.977), LRFS (HR: 3.856, 95% CI: 1.033–14.395), and DMFS (HR: 4.135, 95% CI: 1.401–12.201) between HRG and LRG. However, MRG versus LRG, which is, high EBV DNA or high PLR alone did not show a significant difference in prognosis. This result seems to be inconsistent with previous studies, which may be due to the small sample size of the present study compared to previous studies, and it may be also because the development of NPC is a long process, and the follow-up time of this study is insufficient. Other prognostic factors include sex, age, N stage and histology type. In the analysis of ROC curve, the AUC value of EBV DNA-PLR was higher than that of EBV DNA and PLR on 3-year OS, LRFS and DMFS, which showed some advantages in predicting prognosis of NPC patients.

There are some other limitations in this study, such as the EBV DNA-PLR shown in the results is an independent risk factor for 3-year OS (HR: 11.897, 95% CI: 2.441–57.977) in NPC patients, but the 95% confidence interval of HR is wide. And there is not any statistically significant difference in the ROC curve of EBV DNA-PLR, EBV DNA and PLR. This may be related to the insufficient number of final events due to the small sample size. In addition, this is a retrospective study with a relatively short follow-up period and a lack of 5-year survival results, which may affect the correct prediction of long-term outcomes. Then, the treatment of all eligible patients varied according to the choice of doctors in charge, and there are differences in chemotherapy regimens, chemotherapy cycles and drug doses, which may affect the results of the study. In the future, we will need to expand the sample size, further control other factors that may affect the results, and explore a more complete scoring system to better guide clinical diagnosis and treatment.

## Conclusion

In summary, the EBV DNA-PLR combined score could be used as an individualized clinical assessment tool to more accurately and easily predict the prognosis of patients with non-metastatic NPC.

## Data Availability

The datasets used and analysed during the current study are available from the corresponding author on reasonable request.

## Declarations

### Abbreviations

EBV, Epstein-Barr virus; PLR, platelet-to-lymphocyte ratio; NLR, neutrophil-to-lymphocyte ratio; CRP, C-reactive protein; LDH, lactate dehydrogenase; NPC, nasopharyngeal carcinoma; OS, overall survival; LRFS, local recurrence-free survival; DMFS, distant metastasis-free survival; HRG, high risk group; MRG, medium risk group; LRG,low risk group; ROC, receiver operating characteristic; AUC, area under the curve; IMRT, intensity-modulated radiotherapy; CCRT, concurrent chemoradiotherapy; IC, induction chemotherapy; AC, adjuvant chemotherapy; HR, hazard ratio; CI, confidence interval.

**Ethics approval and consent to participate**.

This study was conducted in accordance with the Helsinki Declaration and approved by the Ethics Committee of the First Affiliated Hospital of Guangxi Medical University. And this was a retrospective study, so the informed consent was waived by the Ethics Committee of the First Affiliated Hospital of Guangxi Medical University. Participant information is confidential.

**Consent for publication**.
